# Association of shift work and dietary inflammatory potential with all-cause death among us hypertensive population: national health and nutrition examination study, 2005–2010

**DOI:** 10.1186/s12889-023-15740-6

**Published:** 2023-06-06

**Authors:** Yukun Li, Xiaodong Peng, Xuesi Wang, Rong Lin, Xinmeng Liu, Fanchao Meng, Xiaoying Liu, Linling Li, Rong Bai, Songnan Wen, Yanfei Ruan, Ribo Tang, Nian Liu

**Affiliations:** 1 Department of Cardiology, Beijing Anzhen Hospital, Capital Medical University, Beijing, 100012 China; 2 National Clinical Research Center for Cardiovascular Diseases, Beijing, 100012 China; 3 Banner University Medical Center Phoenix, College of Medicine University of Arizona Phoenix, Arizona, 85123 USA; 4 North China Medical & Health Group XingTai Genernal Hospital, Xingtai, 054000 China; 5 Department of Cardiology, Bejing Chuiyangliu Hospital, Beijing, 100012 China

**Keywords:** Public health, Shift work, Dietary inflammatory potential, Mortality risk, Hypertensive population, Occupational health.

## Abstract

**Background & aims:**

The individual effect of working schedule on survival in the hypertensive population has not been adequately studied. Shiftworkers are also prone to unhealthy lifestyles like pro-inflammatory diet. Therefore, we assessed the effect of shift work and its joint association with dietary inflammatory potential on mortality risk among the large US nationally representative sample of adult hypertensive population.

**Methods:**

Data were from a nationally representative prospective cohort among US hypertensive population (n = 3680; weighted population, 54,192,988). The participants were linked to the 2019 public-access linked mortality archives. The working schedule were self-reported using the Occupation Questionnaire Section. Dietary inflammatory index (DII) scores were equally calculated using the 24-hour dietary recall (24 h) interviews. Multivariable Cox proportional hazards regression models were used to estimate hazard ratio and 95% confidence intervals (95%CI) for survival of hypertension individuals by work schedule and dietary inflammatory potential. The joint effect of work schedule and dietary inflammatory potential was then examined.

**Results:**

Among the 3680 hypertension individuals (39.89% female [n = 1479] and 71.42% white [n = 1707]; weighted mean [SE] age, 47.35 [0.32] years), 592 individuals reported shift work status. 474 (10.76%) reported shift work status with pro-inflammatory dietary pattern (DII scores > 0). 118 (3.06%) reported shift work status with anti-inflammatory dietary pattern (DII scores < 0). 646 (19.64%) reported a non-shift working schedule with anti-inflammatory dietary pattern, while 2442 (66.54%) reported non-shift working schedule with pro-inflammatory dietary pattern. After a median follow-up of 11.67 years (140 months), 317 deaths (cardiovascular diseases (CVD), 65; cancer, 104) were registered. Cox regression analysis showed that shift work was associated with higher risk of all-cause mortality (hazard ratio [HR], 1.48; 95% CI, 1.07–2.06) compared with non-shift workers. In the joint analysis, shift work status combined with pro-inflammatory dietary pattern was associated with the highest all-cause mortality risk. Moreover, adopting the anti-inflammatory diet significantly attenuates the deleterious effect of shift work on mortality risk.

**Conclusions:**

In this large representative sample of adults with hypertension in the U.S., the combination of shift work status with pro-inflammatory dietary pattern was highly prevalent and was associated with the highest risks of death from all causes.

## Introduction

Hypertension is a major global health problem and remains the single largest contributor to the burden of disease and all-cause mortality [1]. The prevalence of hypertension is on a rapid increase and it nearly doubled between 1990 and 2019 [2]. According to recent data from the Global Burden of Disease (GBD) 2020, increased blood pressure results in over ten million deaths annually worldwide [3]. Different causes of death (COD) were found to be closely associated with hypertension such as cardiovascular disease, kidney disease, pulmonary disease, neurological disease, infection, cancer, and cerebrovascular accident-related deaths [4–7]. Long-term effect of hypertension can shorten the life expectancy. Unhealthy dietary pattern such as high fat/high sugar diet as well as occupational exposures like shift work/noise are both strongly involved with the onset, development, and prognosis of hypertension [8–10]. Therefore, timely evaluation and intervention of the above risk factors is key to alleviating the disease burden and mitigating the mortality risk among patients with hypertension.

Shift work commonly refers to all kinds of work not scheduled in daytime. With the acceleration of urbanization and refinement of technical and social division of labor, the shiftwork status has become prevalent in modern society. According to the US Bureau of Labor Statistics, nearly 16% of the workforce worked in shifts, identified as an common occupational exposure as well as irregular working schedule [11]. The shiftwork status brings about a series of unwanted adverse effects such as sleep deprivation, melatonin secretion disorders, disorganized immune/circadian systems and dysregulated immune processes [12–14]. The aforementioned negative impacts of shiftwork were strongly associated with a rise of all-cause and cause-specific mortality risk [15–18]. Moreover, the increasing number of epidemiological studies have indicated the potential causality between shift work and increased risk of death in the general population and specific occupational groups like nurses and chemical workers [19–21]. However, there are no studies evaluating the influence of shift work on the mortality risk of patients with hypertension till date.

As varying compositions of food possess corresponding anti-inflammatory or pro-inflammatory properties, diet is considered an important factor in the regulation of chronic systemic inflammation [22], which has been identified as a risk factor for mortality across populations. The Dietary Inflammation Index (DII), a literature-derived validated tool encompassing 45 items, was developed to better quantitatively evaluate the inflammatory potential of various nutrients in daily meals. Positive values of DII scores represent a pro-inflammatory dietary pattern, while negative values represent an anti-inflammatory dietary pattern [23]. Previous studies revealed that shiftwork status is independently associated with an increased level of systemic inflammatory indicators [24]. The higher mean value of DII scores (indicating more pro-inflammatory dietary pattern) and more serious food insecurity were also observed in shift workers [25, 26], which explains the augmented risk of inflammation-related chronic disease in this population.

The causal relationship between hypertension and inflammation has been explored for many years, and hypertension is considered a multi-factorial chronic inflammatory disease to some extent [27]. Recent studies have shown that the DII scores were positively associated with systolic blood pressure (SBP) values and prevalence of hypertension, and patients previously diagnosed with cardiovascular disease also tend to have a pro-inflammatory dietary pattern [28–30]. Several novel therapeutic strategies exerting anti-inflammatory and immune-modulating effects improved the long-term prognosis of the hypertensive population via reducing end-organ damage [31, 32]. The relationship between DII score and risk of death was reported in a series of studies, in which pro-inflammatory dietary pattern was confirmed to be linked with higher mortality risk [33, 34]. However, epidemiologic evidence remains missing on the joint association of shift work and dietary inflammatory potential with survival in hypertensive population who may be restricted in dietary consumption.

In general, this study aimed to examine the effect of shift work and its joint association with dietary inflammatory potential on mortality risk in a large U.S. nationally representative sample of the adult hypertensive population. The findings generated are beneficial to lifestyle modifications and protection of occupational health among hypertensive population.

## Materials and methods

### Sample and population

Analysis of this study were performed under the NHANES analytic guidelines, which is conducted by the National Center for Health Statistics of the Centers for Disease Control and Prevention, Maryland. The NHANES employs a stratified multi-stage sampling design. Details of sampling and testing process have been well outlined in previously published articles. Briefly, systematic health-related interviews and examinations were conducted in 2-year cycles. The survey included participants from different geographical locations and racial/ethnic groups to guarantee its representativeness. NHANES protocols have been approved by the National Center for Health Statistics research ethics review board, and written informed consent was obtained from all enrolled participants.

Data of our study were collected from three cycles of NHANES (2005–2010). In the present study, hypertension was diagnosed among those who responded yes to question of self-reported hypertension (“Have you ever been told by a doctor or other health professional that you had hypertension, also called high blood pressure?”); or use of anti-hypertension drugs (“Because of your high blood pressure/hypertension, have you ever been told to take prescribed medicine?”); or the abnormal average value of three blood pressure measurements (systolic blood pressure greater than 130 mm Hg or diastolic blood pressure greater than 80 mm Hg).

Question (OCQ265) entitled “which of the following best describes the hours you usually work at your main job or business?” in Occupational Questionnaire Section (OCQ) was used to evaluate the shiftwork status. The categorization of shiftwork status was established according to the answer of OCQ265 : (1) a regular daytime schedule, (2) a regular evening shift, (3) a regular night shift, (4) a rotating shift, and (5) another schedule. As the answer (5) lacked further clarification, participants who answered “another schedule” were removed. We treat participants with evening/night shift or rotating shift work as “shift workers” group, and those with a regular daytime schedule was referred to “non-shift workers” group.

**Fig. 1:**
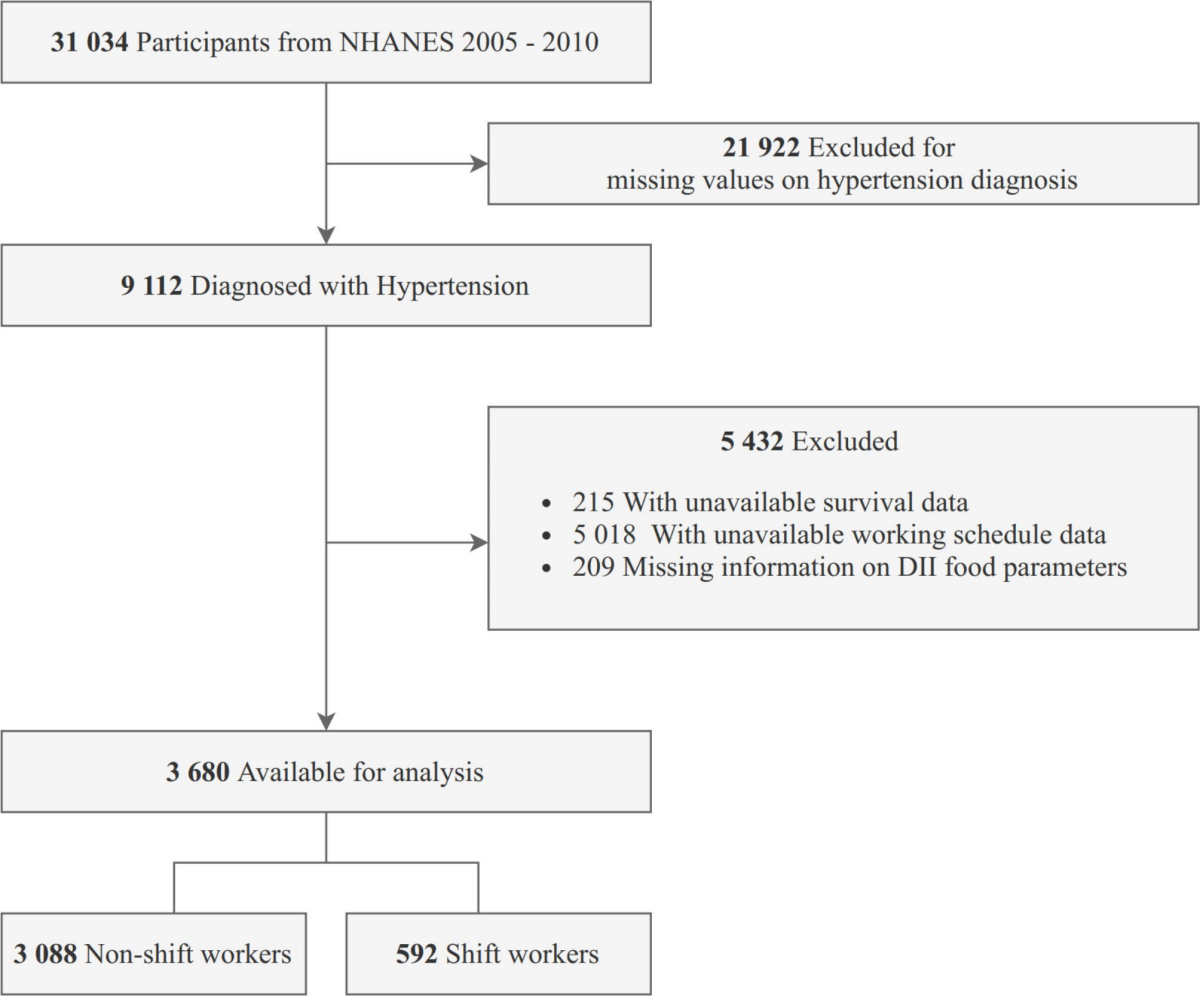
Flowchart Diagram of the Screening and Enrollment of Study Participants

Hypertensive adults with assessment of shiftwork status, DII scores, and survival data (details are described below) were enrolled. After excluding those with missing data, 3680 participants were included in the final analysis. The flowchart of participant enrollment is shown in Fig. 1.

### Dietary inflammatory index and shiftwork exposures

Shivappa et al. first introduced the DII as a novel and effective indicator to assess the inflammatory potential of daily diet [23]. The DII is based on the summary of nearly 2,000 research articles published from 1950 to 2010. Its scores reflect the inflammatory effect of 45 nutrients, foods, and other dietary bioactive compounds on six inflammatory biomarkers (C-Reactive Protein, TNF-α, IL-1β, IL-4, IL-6, and IL-10). Methods to calculate DII score have been previously described in many articles. Daily dietary information was obtained by 24-hour dietary recalls (24 h). Each food item was then assigned an overall inflammatory effect score. After subtracting the standard means intake of each parameter, results of all food items were divided by their standard deviation (SD). These values were converted into a centered percentile score and then multiplied by a corresponding food-parameter inflammatory effect score. The DII score is sum of all the above food parameters. Negative scores of DII score indicate an anti-inflammatory diet and positive scores indicates a pro-inflammatory diet [35]. The validity of DII has been verified in a number of previous studies based on data from NHANES or other databases [36–39].

We assessed shift work status via the participants’ answers from Occupation Questionnaire Section. The corresponding question entitled “Which of the following best describes the hours you usually work at your main job or business?” Optional answers included: (1) regular daytime, (2) evening shifts, (3) night shifts, (4) rotating shifts, or (5) another schedule. For the aim of our analysis, a participant was considered as a shift worker if he reported working evening/night shift or rotating shift schedule, and all other participants were defined as the comparison group.

### Mortality data

Mortality data were derived from the 2019 public-access linked mortality archives through December 31, 2019. It was matched with files from the National Death Index using a probabilistic matching algorithm and all adult participants of NHANES were accessible for mortality follow-up. We analyzed mortality from all causes, CVD (codes I00-I09, I11, I13, and I20-I51), cancer(codes C00-C97) and other causes.

### Covariates

Age, gender, ethnicity/race (non-Hispanic white, Mexican American, non-Hispanic black, other Hispanic and others(including multi-racial)), educational level (less than high school, high school or equivalent, and college or above), and socioeconomic status (poverty to income ratio, PIR = Family income / Poverty threshold for family size and composition) were derived from interviews and physical examinations. Metabolic equivalent (MET) measures energy metabolism level during different activities. Physical activity was assessed via the Physical Activity Questionnaire (PAQ) section and calculated using the formula: PA(MET-h/wk) = MET *weekly frequency* duration of each of PA. Different MET values were assigned for various types of physical activity by NHANES, vigorous work-related activity(MET = 8.0), vigorous leisure-time physical activity(MET = 8.0), moderate work-related activity(MET = 4.0), walking or bicycling for transportation(MET = 4.0), and moderate leisure-time physical activity(MET = 4.0). Participants were assigned to three subgroups: no PA(PA < 1MET-h/wk), low intensity PA(PA = 1-48MET-h/wk), and high intensity physical activity(PA > 48 MET-h/wk) based on their PA scores.

Smoking status was classified as “never” (smoked less than 100 cigarettes in life), “former” (smoked more than 100 cigarettes in life and smoke not at all now), “now” (smoked moth than 100 cigarettes in life and smoke some days or every day). Status of alcohol use were grouped into (1) never (< 12 drinks in lifetime), (2) former (≥ 12 drinks in 1 year and did not drink last year, or did not drink last year but drank ≥ 12 drinks in lifetime), (3) current mild alcohol use(<2 drinks per day for women, <3 drinks per day for men), (4) current moderate alcohol use (≥ 2 drinks per day for women, ≥ 3 drinks per day for men, or binge drinking ≥ 2 days per month), (5) current heavy alcohol use (≥ 3 drinks per day for women, > 4 drinks per day for men, or binge drinking on 5 or more days per month). The value of body mass index (BMI) was calculated as weight (kg) divided square of height (m^2^), and was divided into three groups according to the cut-off value of 25 and 30 (BMI ≥ 30 is defined as obese). Family income-poverty ratio (PIR) was classified into three groups: < 1.30, 1.30–3.49, ≥ 3.5. History of hyperlipidemia, diabetes, CVD, or depression (PHQ9 ≥ 10) were also derived from questionnaires. Multiple imputation was used for missing values of covariates.

### Statistical analysis

All analysis accounted for the complex stratified survey design and NHANES sampling weights under the NHANES analytic guidelines in order to ensure nationally representative estimates. Continuous variables of the baseline characteristics in our study were reported with means and SDs and weighted percentages was used for categorical variables. Statistical tests were 2-sided and statistical significance was assigned as p < 0.05. Data analyses were conducted from April 1 to July 1, 2022, using R and R Studio (R Foundation for Statistical Computing, R Version 4.2.0). Multivariable Cox proportional hazards regression models were used to compute the hazard ratios (HR) and 95% CI were used for associations of shift work and all-cause, CVD-specific, cancer-specific and other (non-cancer/non-CVD) causes of mortality. The fully adjusted multivariable model were adjusted for age, gender, race and ethnicity, educational level, physical activity, family poverty income ratio, BMI, smoking status, alcohol use, and health conditions (hyperlipidemia, depression, and history of diabetes and/or CVD). To assess the effect of dietary inflammatory patterns, participants were grouped according to working schedule (shift worker/non-shift worker) and DII scores (pro/anti-inflammatory diet) via multi-variable Cox proportional hazards regression models adjusting for the same covariates to estimate corresponding mortality risks. All analyses were performed in overall population and in women, men, obese (BMI ≥ 30) and nonobese (BMI<30) subgroups respectively. Lastly, the sensitivity analysis excluding deaths that occurred during the first 2 years of follow-up was performed in order to lessen the potential for reverse causation.

## Results

Among the 3680 hypertensive individuals (weighted population, 54,192,988; weighted mean [SE] age, 47.35 [0.32] years; 39.89% female) in the study cohort, 1707 (71.42%) were Non-Hispanic White, 616 (6.77%) were Mexican American, 917 (12.66%) were Non-Hispanic Black, 286 (3.67%) were Other Hispanics, and 154 (5.49%) were individuals of other races, including American Indian/Native Alaskan/Pacific Islander, Asian, and multiracial. The baseline profile of the participants stratified according to shiftwork status are shown in Table 1. Shift workers tended to be younger[44.27 vs. 47.85], black[206 (20.75) vs. 711 (11.36)], High school graduation[260 (43.33%) vs. 1173 (33.45%)], low-income[164(18.65) vs. 576(10.80)], and were current smokers [164(27.14) vs. 615(19.16)]. Other characteristics did not differ between the non-shift workers group and shift worker group.

**Table 1:** Sample Size^a^ and Characteristics of US hypertensive population, NHANES 2005 to 2010

Variable	Study population		
	All	Non-shift workers	Shift workers
Overall	3680	3088	592
Age(years)	47.35 ± 0.32	47.85 ± 0.33	44.27 ± 0.51
sex			
Women	1479(39.89)	1261(40.37)	218(36.92)
Men	2201(60.11)	1827(59.63)	374(63.08)
Race and ethnicity			
Non-Hispanic White	1707(71.42)	1500(73.24)	207(60.09)
Mexican American	616(6.77)	518(6.65)	98(7.53)
Non-Hispanic Black	917(12.66)	711(11.36)	206(20.75)
Other Hispanic	286(3.67)	231(3.35)	55(5.64)
Other Race - Including Multi-Racial	154(5.49)	128(5.41)	26(5.98)
Educational attainment			
<High school	315(4.20)	257(4.08)	58(4.90)
=High school	1433(34.81)	1173(33.45)	260(43.33)
>High school	1932(60.99)	1658(62.47)	274(51.77)
Physical activity			
No PA	138(3.75)	108(3.39)	30(5.48)
Low intensity PA	2180(59.24)	1890(64.78)	290(51.48)
High intensity PA	1362(37.01)	1090(31.83)	272(43.04)
Family poverty income ratio			
< 1.30	740(11.89)	576(10.80)	164(18.65)
1.30–3.49	1366(32.81)	1102(31.12)	264(43.29)
>=3.5	1574(55.31)	1410(58.07)	164(38.06)
Weight status, BMI			
< 25	706(20.18)	596(20.44)	110(18.54)
25–30	1229(33.74)	1049(34.09)	180(31.54)
>=30	1745(46.08)	1443(45.46)	302(49.92)
Smoking			
Never	1986(53.96)	1674(54.16)	312(52.78)
Former	915(25.77)	799(26.68)	116(20.09)
Now	779(20.27)	615(19.16)	164(27.14)
Alcohol use			
Never	368(8.08)	306(7.96)	62(8.85)
Former	633(14.83)	534(14.92)	99(14.32)
Mild	1253(37.79)	1072(38.65)	181(32.47)
Moderate	565(15.94)	478(15.92)	87(16.03)
Heavy	861(23.35)	698(22.56)	163(28.33)
Hyperlipidemia			
No	918(22.84)	754(22.47)	164(25.13)
Yes	2762(77.16)	2334(77.53)	428(74.87)
Diabetes			
No	3226(90.38)	2709(90.64)	517(88.76)
Yes	454(9.62)	379( 9.36)	75(11.24)
Cardiovascular disease			
No	3427(93.64)	2875(93.61)	552(93.78)
Yes	253(6.36)	213(6.39)	40(6.22)
Depression, PHQ9			
[0,9]	3474(95.24)	2924(95.39)	550(94.31)
[10,27]	206(4.76)	164(4.61)	42(5.69)

BMI, Body Mass Index (calculated as weight in kilograms per metre squared.)

PHQ9, Patient Health Questionnaire-9

NHANES, the National Health and Nutrition Examination Survey

^a^ Weighted to be nationally representative. The weighted percentage may not sum to 100% due to missing data

**Fig. 2:**
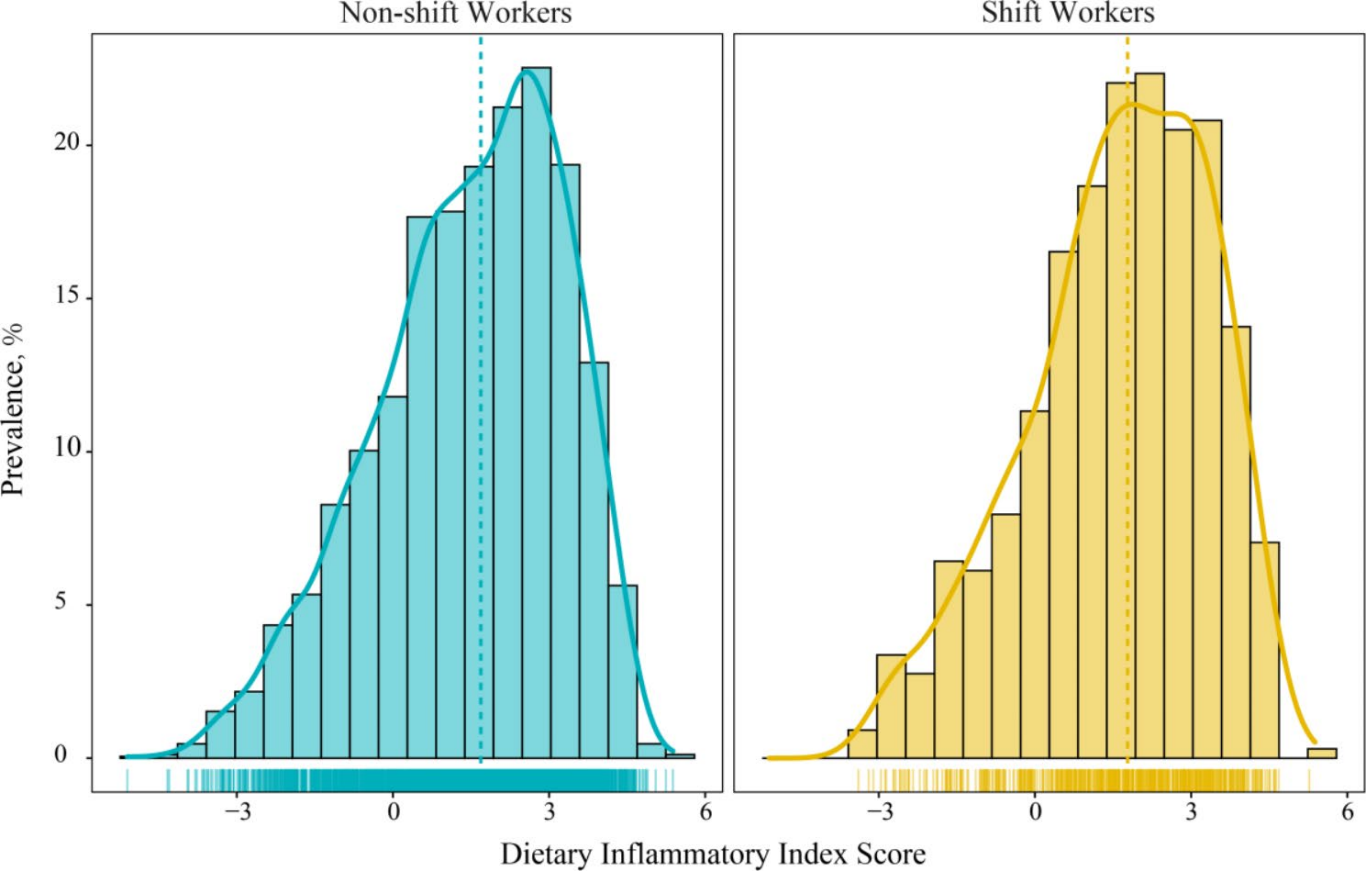
The Distribution of Dietary Inflammatory Index Score in Shift Workers and Non-shift Workers

The distribution of DII scores is displayed in Fig. 2. The corresponding median DII scores were 1.69 (0.31–2.85) and 1.78(0.53–2.96) in hypertensive non-shift workers and shift workers.

**Fig. 3:**
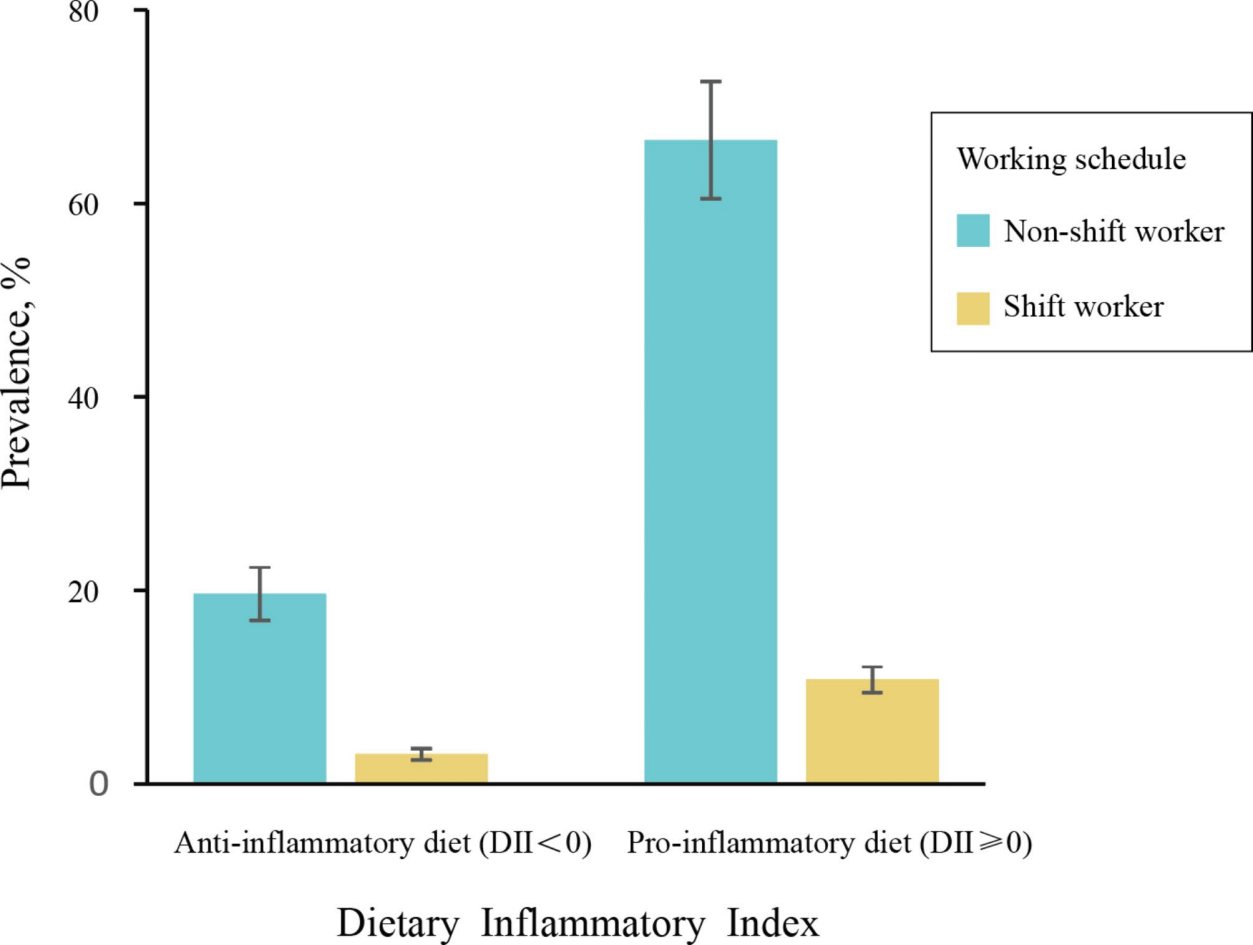
Joint Prevalence of Work Schedule and Dietary Inflammatory Potential Among US hypertensive population, NHANES 2005 to 2010 Data were weighted to be nationally representative and accounted for the complex sampling design. Error bars indicate 95% CIs. NHANES refers to the National Health and Nutrition Examination Survey

The prevalence of hypertensive population stratified according to working schedule and dietary inflammatory potential are shown in Fig. 3. Only 3.06% of the hypertensive patients reported shiftwork status as well as following the anti-inflammatory diet (DII<0), while 10.76% reported pro-inflammatory dietary pattern as well as being shift workers. Even in non-shift workers population, consuming pro-inflammatory diet was more prevalent (66.54% vs. 19.64%).

Among 3680 participants, a total of 317 deaths occurred during a median follow-up of 11.67 years (140 months), including 65 deaths from cardiovascular diseases (CVD) and 104 deaths from cancer. Table 2 demonstrates the association of shift work with total and cause-specific mortality. Significant positive relationships were found between shiftwork status and total/other mortality in hypertensive shift workers, but not for CVD and cancer mortality. The HR and 95% CI for all cause and non-cardiac or cancer-specific mortality were 1.61 (1.14,2.28) and 1.83 (1.09,3.05) in the minimally-adjusted model. After further adjusting for covariates in fully adjusted model, HRs for all-cause, non-cardiac or cancer specific mortality among hypertensive shift workers were 1.48 (95% CI, 1.07–2.06) and 1.64 (95% CI, 1.01–2.68), respectively.

**Table 2:** Association of Work Schedule With All-Cause, CVD, Cancer, and Other Mortality Among US hypertensive population, NHANES 2005 to 2010

Mortality Outcome	Death/No.	Weighted Death(%)	Hazard Ratio(95%)	
			Minimally adjusted model	Fully adjusted model
All Cause				
Current work schedule				
Non-shift Workers	263/3088	3129685.61(6.70)	1 [Reference]	1 [Reference]
Shift Workers	54/592	668820.63(8.93)	1.61(1.14,2.28)	1.48(1.07,2.06)
CVD				
Current work schedule				
Non-shift Workers	54/3088	597583.02(1.28 )	1 [Reference]	1 [Reference]
Shift Workers	11/592	141197.06(1.89)	1.84(0.80,4.25)	1.80(0.82,3.92)
Cancer				
Current work schedule				
Non-shift Workers	89/3088	1068210.91(2.29)	1 [Reference]	1 [Reference]
Shift Workers	15/592	168578.13(2.25)	1.18(0.59,2.36)	1.13(0.58, 2.22)
Others				
Current work schedule				
Non-shift Workers	120/3088	1463891.67(3.13)	1 [Reference]	1 [Reference]
Shift Workers	28/592	359045.44(4.79)	1.83(1.09,3.05)	1.64(1.01,2.68)

Minimally adjusted model, we adjusted for age, sex, race/ethnicity, education attainment

Fully adjusted model, we futher adjusted for physical activity, family poverty income ratio, BMI, smoking status, alcohol use, Hyperlipidemia, Diabetes, Cardiovascular disease, PHQ9.

The joint analysis of work schedule and dietary inflammatory patterns revealed that among hypertensive population, shift workers with pro-inflammatory dietary pattern was associated with the highest all-cause mortality risk. However, shift workers with anti-inflammatory dietary pattern was not associated with increased all-cause mortality risk (Table 3).

**Table 3:** Joint Association of Work Schedule and Dietary Inflammatory Potential With All-Cause Mortality Among US hypertensive population, NHANES 2005 to

Category	Death/No.	Weighted Death(%)	Hazard Ratio(95%)	
			Minimally adjusted model	Fully adjusted model
Non-shift Workers with anti-inflammatory diet	42/646	566265.87(5.32)	1 [Reference]	1 [Reference]
Non-shift Workers with pro-inflammatory diet	12/118	128194.56(7.72)	1.47(1.05,2.04)	1.30(0.92,1.84)
Shift Workers with anti-inflammatory diet	221/2442	2563419.73(7.11)	1.61(0.61,4.27)	1.57(0.65,3.83)
Shift Workers with pro-inflammatory diet	42/474	540626.07(9.28)	2.40(1.47,3.91)	1.92(1.21,3.05)
P for trend			< 0.001	0.004

Minimally adjusted model adjusted for age, sex, race/ethnicity, education attainment

Fully adjusted model, we futher adjusted for physical activity, family poverty income ratio, BMI, smoking status, alcohol use, Hyperlipidemia, Diabetes, Cardiovascular disease, PHQ9.

Comparing the hazard ratio of shift workers (anti-inflammatory diet) [HR = 1.57, 95%CI 0.65–3.83] with those of non-shift workers (anti-inflammatory diet), it is found that anti-inflammatory dietary pattern partially moderated and attenuated the adverse effect of shift work on all-cause mortality risk. Whereas, the significant increase of mortality risk among shift workers receiving pro-inflammatory diet [HR = 1.92, 95%CI 1.21–3.05] indicated the joint effect of pro-inflammatory dietary pattern and shift work on mortality risk.

**Fig. 4:**
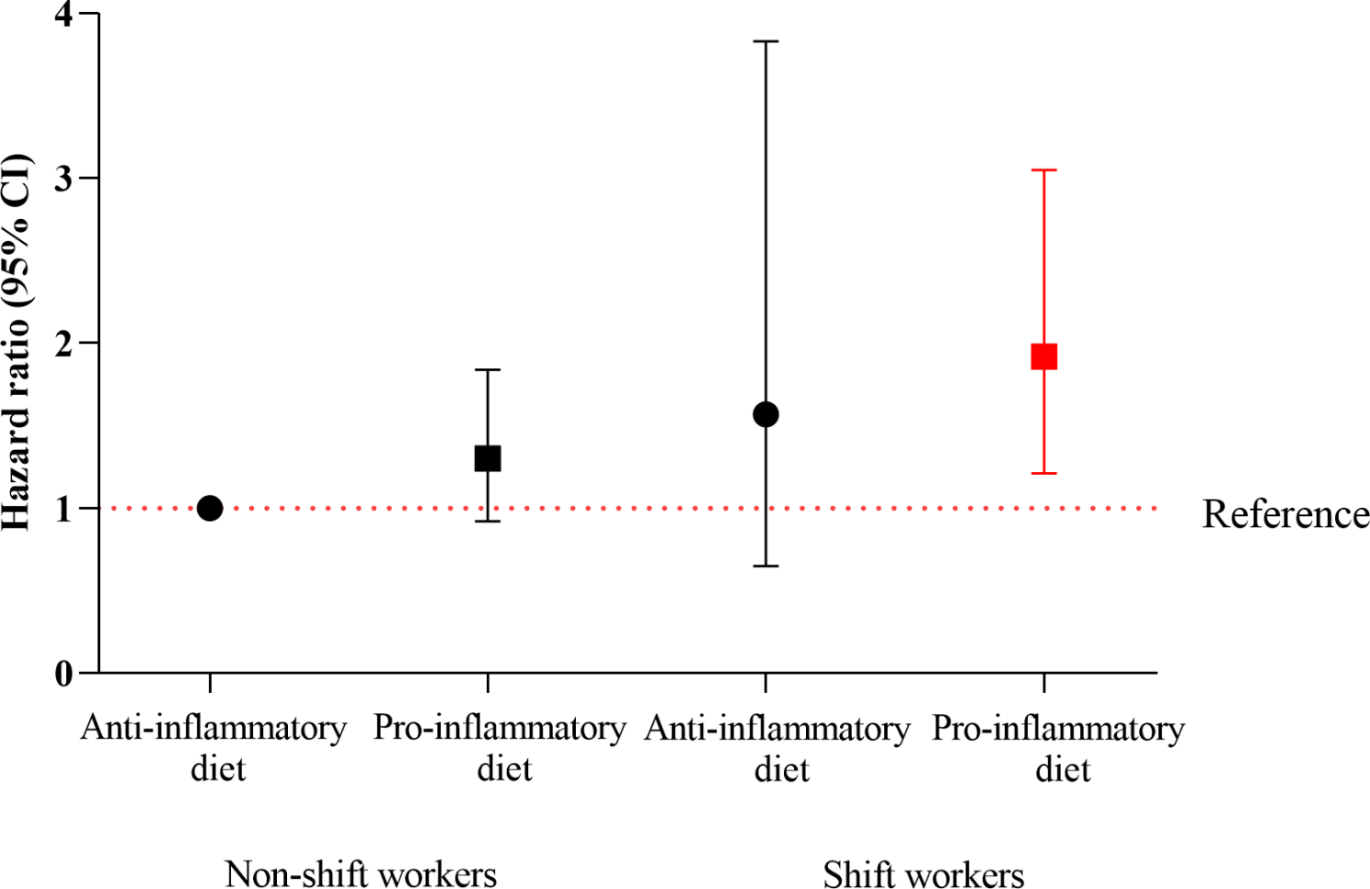
Joint Association of Work Schedule and Dietary Inflammatory Potential With All-Cause Mortality Among US hypertensive population, NHANES 2005 to 2010 Hazard ratios(solid symbols) with 95% CIs(error bars) of joint categories of work schedule and dietary inflammatory potential for all-cause mortality were estimated via weighted multivariable Cox regression models adjusted for age, sex, race/ethnicity, education attainment, physical activity, family poverty income ratio, BMI, smoking status, alcohol use, Hyperlipidemia, Diabetes, Cardiovascular disease, PHQ9. BMI refers to body mass index(calculated as weight in kilograms per metre squared); PHQ9 refers to patient health questionnaire-9 and NHANES, the National Health and Nutrition Examination Survey

Figure 4 visually demonstrates the association of shift work and dietary inflammatory patterns on the primary outcomes. Only the combinations of pro-inflammatory diet and shift work were deleteriously associated with rising mortality risk of hypertensive individuals.

Tables 4 and 5 show results stratified by sex and baseline BMI. The impact of shiftwork status on all-cause mortality was stronger in obese individuals than in nonobese individuals, as well as in male adults. Associations of shiftwork and DII with all-cause mortality were unaltered between sexes. All results remained similar in sensitivity analyses excluding deaths that occurred during the first 2-year follow-up (Table 6).

**Table 4:** Association of Work Schedule With All-Cause Mortality Among US Hypertensive Population by Sex and Obesity, NHANES 2005 to 2010

Subgroup	Category	Death/No.	Weighted Death(%)	Hazard Ratio(95%)	
				Minimally adjusted model	Fully adjusted model
Sex					
Female	Non-shift Workers	81/1261	975769.92(5.18)	1 [Reference]	1 [Reference]
	Shift Workers	19/218	201685.38(7.29)	1.79(0.96,3.32)	1.70(0.89,3.21)
Male	Non-shift Workers	182/1827	2153915.68(7.73)	1 [Reference]	1 [Reference]
	Shift Workers	35/374	467135.25(9.89)	1.59(1.03,2.46)	1.59(1.08,2.36)
P for interaction				0.81	0.93
Obesity					
No	Non-shift Workers	158/1645	1879698.73(7.38)	1 [Reference]	1 [Reference]
	Shift Workers	29/290	341298.91(9.10)	1.44(0.75,2.76)	1.57(0.83,2.98)
Yes	Non-shift Workers	105/1443	1249986.87(5.89)	1 [Reference]	1 [Reference]
	Shift Workers	25/302	327521.73(8.76)	1.93(1.17,3.18)	1.74(1.03,2.94)
P for interaction				0.60	0.76

Minimally adjusted model adjusted for age, sex, race/ethnicity, education attainment

Fully adjusted model, we futher adjusted for physical activity, family poverty income ratio, BMI, smoking status, alcohol use, Hyperlipidemia, Diabetes, Cardiovascular disease, PHQ9.

**Table 5:** Joint Association of Work Schedule and Dietary Inflammatory Potential With All-Cause Mortality Among US hypertensive population by Sex and Obesity, NHANES 2005 to 2010

Subgroup	Category	Death/No.	Weighted Death(%)	Hazard Ratio(95%)	
				Minimally adjusted model	Fully adjusted model
Sex					
Female	Non-shift Workers with anti-inflammatory diet	4/175	53985.35(1.85)	1 [Reference]	1 [Reference]
	Non-shift Workers with pro-inflammatory diet	77/1086	921784.58(5.78)	3.45(1.27, 9.39)	3.36(1.28, 8.81)
	Shift Workers with anti-inflammatory diet	3/25	21028.98(7.10)	3.76(0.83,17.14)	3.42(0.84,14.00)
	Shift Workers with pro-inflammatory diet	16/193	180656.40(7.32)	5.76(1.69,19.57)	5.41(1.58,18.56)
Male	Non-shift Workers with anti-inflammatory diet	38/471	512280.52(6.63)	1 [Reference]	1 [Reference]
	Non-shift Workers with pro-inflammatory diet	144/1356	1641635.16(8.16)	1.21(0.86,1.69)	1.11(0.76,1.62)
	Shift Workers with anti-inflammatory diet	9/93	107165.58(7.85)	1.37(0.47,4.00)	1.48(0.56,3.93)
	Shift Workers with pro-inflammatory diet	26/281	359969.67(10.71)	2.06(1.19,3.57)	1.83(1.10,3.02)
P for interaction				0.16	0.23
Obesity					
No	Non-shift Workers with anti-inflammatory diet	26/367	344167.85(5.51)	1 [Reference]	1 [Reference]
	Non-shift Workers with pro-inflammatory diet	132/1278	1535530.88(7.99)	1.41(0.87,2.29)	1.40(0.86,2.28)
	Shift Workers with anti-inflammatory diet	8/63	99187.66(10.90)	2.01(0.49,8.36)	1.99(0.51,7.67)
	Shift Workers with pro-inflammatory diet	21/227	540626.07(9.28)	1.87(0.83,4.19)	2.05(0.89,4.69)
Yes	Non-shift Workers with anti-inflammatory diet	16/279	222098.03(5.05)	1 [Reference]	1 [Reference]
	Non-shift Workers with pro-inflammatory diet	89/1164	1027888.85(6.11)	1.65(0.77,3.52)	1.37(0.66,2.82)
	Shift Workers with anti-inflammatory diet	4/55	29006.91(3.86)	1.12(0.29,4.36)	0.90(0.25,3.25)
	Shift Workers with pro-inflammatory diet	21/247	298514.82(9.99)	3.41(1.42,8.21)	2.50(1.11,5.62)
P for interaction				0.67	0.75

Minimally adjusted model adjusted for age, sex, race/ethnicity, education attainment

Fully adjusted model, we futher adjusted for physical activity, family poverty income ratio, BMI, smoking status, alcohol use, Hyperlipidemia, Diabetes, Cardiovascular disease, PHQ9.

**Table 6:** Association of Work Schedule and Dietary Inflammatory Potential With All-Cause Mortality Among US hypertensive population excluding deaths within first 2 years

Subgroup	Death/No.	Weighted Death(%)	Hazard Ratio(95%)	
			Minimally adjusted model	Fully adjusted model
Current work schedule				
Non-shift Workers	242/3067	2880925.21(6.20)	1 [Reference]	1 [Reference]
Shift Workers	52/590	654770.62(8.76)	1.70(1.18,2.44)	1.59(1.13,2.26)
Current work schedule & DII				
Non-shift Workers with anti-inflammatory diet	40/644	527076.26(4.97)	1 [Reference]	1 [Reference]
Non-shift Workers with pro-inflammatory diet	202/2423	2353848.94(6.57)	1.44(1.01,2.05)	1.27(0.87,1.86)
Shift Workers with anti-inflammatory diet	11/117	123433.03(7.45)	1.64(0.60,4.48)	1.66(0.66,4.15)
Shift Workers with pro-inflammatory diet	41/473	531337.59(9.13)	2.50(1.53,4.08)	2.02(1.27,3.22)

Minimally adjusted model adjusted for age, sex, race/ethnicity, education attainment

Fully adjusted model, we futher adjusted for physical activity, family poverty income ratio, BMI, smoking status, alcohol use, Hyperlipidemia, Diabetes, Cardiovascular disease, PHQ9.

## Discussion

In this nationally representative prospective cohort among US hypertensive population, shift work was associated with a higher risk of mortality during a median follow-up of 11.67 years. When further including the factor of dietary inflammatory patterns and considering non-shift workers with anti-inflammatory dietary pattern as a reference, the mortality risk significantly increased in shift-workers with pro-inflammatory dietary pattern. However, in those receiving anti-inflammatory diet, the detrimental impact of shift work on mortality risk was alleviated and unsignificant. Notably, hypertensive shift workers adopting pro-inflammatory dietary pattern (DII scores ≥ 0) doubled their risk for all-cause mortality.

Given long-term effects like circadian disruption and sleep deprivation, shift work status is a potential risk factor for mortality among individuals with hypertension, which is one of the most frequent and common cardiovascular diseases [13, 40]. Previous studies mainly focused on the general or occupation-specific population, but the role of lifestyle changes like dietary modification have not been sufficiently considered in the relationship between shiftwork and mortality risk. Jeanette et al. reported that shiftwork status is closely linked with increased risk of all-cause and cause-specific mortalities in a Danish nurse cohort [21]. In a recent SOLID-TIMI 52 study, overnight shift work was associated with a 1.2-fold increased risk of recurrent cardiovascular events among the enrolled 13,026 patients after acute coronary syndrome [41]. These valuable findings indicate a potential causal relationship between shift work and long-term mortality risk in hypertensive population. However, robust clinical evidence concerning this area and recommended intervention of modifiable risk factors for patients with hypertension are still insufficient. To the best of our knowledge, this is the first study that explores the role of shift work and its joint association with dietary inflammatory potential on the risk of death in a nationally representative prospective cohort of participants with hypertension.

Due to changes in eating frequency, diet timing, and food choices, the diet quality of shift workers poses a great health problem. The dietary patterns in shift workers tend to be unhealthy and are usually presented as higher total energy intake and lower intakes of dietary fiber, folic acid, vitamin B2/C, potassium, and calcium [42, 43]. The long-term unhealthy dietary patterns can lead to a series of human health problems. Reihane et al. showed that unhealthy dietary patterns caused by rotational shift work impede the immune system, manifested as significantly high levels of inflammatory cytokines such as IL-6 and TNF-α among such population [43]. Another systematic review by Van Drongelen demonstrated a strong association between shift work and increased body weight [44]. Dietary intervention is a known effective strategy for maintaining good health among shift workers. Recently, Wu et al. compiled data from 12 studies and found that dietary supplements like Vitamin C and Probiotics are beneficial to daily functioning, emotional regulation and sleep quality of shift workers [45].

From data analysis of NHANES ranging from 2005 to 2010 by Michael et al., higher DII scores were observed in shift workers, indicating that these workers may receive more pro-inflammatory diets [46]. The effect of dietary inflammatory potential in shift workers has been found in hypertension-free adults. In a cross-sectional study using data of NHANES 2005–2012, researchers found that shift work status tends to increase the risk of depression, and diet-related inflammation might play a partial mediating role between shift work and depressive symptoms [47]. To our knowledge, this is the first study elucidating that in shift workers suffering from hypertension, pro-inflammatory dietary pattern can further increase mortality risk by nearly two-fold. However, the adverse effect of shiftwork is attenuated among individuals following anti-inflammatory dietary pattern.

Shift work disrupts the circadian rhythm, impairs sleep quality, and affects work-life balance. In its updated construct of cardiovascular health (Life’s Essential 8), the American Heart Association for the first time integrated “sleep health” into the existing seven foundational factors for cardiovascular health including diet and nicotine exposure [48]. For hypertensive individuals, especially those at high risk of cardiovascular diseases, it is still unknown how shift work impacts mortality risks and long-term prognosis, as well as and how the risks regulated by dietary pattern for effective intervention on their actual daily life. The distinct lack of high-level clinical and epidemiologic evidence makes it difficult for the concept update on occupational protections among hypertensive population. Our analysis fills this previously identified knowledge gaps in the area of hypertension management and occupational protection. The result provides direct evidence of the relationship between shift work status and higher risk of death in hypertensive patients, particularly in those with pro-inflammatory dietary pattern. More importantly, these findings suggest that the anti-inflammatory dietary pattern can attenuate the mortality risk due to shift work. As further note, this study was a population-based analysis, which involved the nationally representative cohort of American hypertensive population with a large sample size and sufficiently long follow-up period.

Several biological and sociological factors could account for these findings. On one hand, hypertension has been regarded as a multifactorial disease and vascular inflammation is involved in its pathogenesis and subsequent target organ damage [49, 50]. On the other hand, shift workers are at higher risk of cardiovascular disease such as hypertension and are considered as a vulnerable group from an occupational health perspective [51, 52]. Sleep deprivation, working stress, fatigue, as well as family disruption commonly observed among shift workers contributes to their unhealthy dietary pattern, resulting in a potential increase in the inflammatory nature of their daily diet.

Some other hypothesized biological alterations need to be accounted for in this phenomenon. The classical theories suggest that circadian rhythms in the human body regulate key metabolic and physiological pathways in all tissues [53, 54]. With long-term exposure to light during working hours, the physiological day-night rhythm of shift workers is disrupted, resulting in a reduced melatonin production and circadian disruption. The stress system can be activated chronically in case of constant day-night reversal, thereby suppressing the thyroid, gonadal, and growth hormone axes, and engendering a series of comorbidities such as obesity, hypertriglyceridemia, and hypercholesterolemia [55]. Moreover, stress stimulates the immune system to release pro-inflammatory agents, thereby exerting synergistic effects with the pro-inflammatory diet to worsen prognosis of patients with hypertension [56, 57]. On the contrary, anti-inflammatory dietary pattern exerts a protective role on shift workers via effectively suppressing inflammatory response in the body, which partially explains the worst survival outcomes among shift workers with pro-inflammatory dietary pattern and relatively good prognosis in hypertensive shift workers following anti-inflammatory diet. Lastly, shift work has been found to break the pro-oxidant/anti-oxidant balance and induce the upregulated oxidative stress and mitochondrial dysfunction [58]. The present analysis showed that the shift work-mortality association was stronger in the male and obese subgroup. Shift work related oxidative stress explains the differences between subgroups, and increasing evidence indicates that the antioxidant activity differs among men and women as well as nonobese and obese individuals [59–62]. Accordingly, more attention should be paid to the higher risk of shift work in male and obese adults with hypertension. Future studies are urgently needed to confirm the causality of this relationship. Moreover, evidence-based interventions can be integrated into the management of patients with hypertension via adjusting working schedule and dietary pattern.

### Strengths and limitations

This is the first study that examines the association of shift work and mortality in NHANES, a nationally representative cohort with a large sample from the well-designed nationwide investigation in the U.S., which adjusted for some covariates including demographic characteristics, health condition, and complete death records.

However, we encountered some limitations. The most fundamental limitation of our study is the high risk of residual confounding. DII score, the information of dietary inflammatory potential, was based on self-report from 24-hour dietary recall that could ignore important aspects of day-to-day variability in diet and recall bias to some extent. In addition, the covariates regarding comorbidities and health indicators were collected at a single time point, but these information can alter over time during the follow-up. Moreover, this study is limited by the relatively small sample size considering number of covariates. Thus, conclusion should be interpreted cautiously. Finally, shift work status and DII scores were assessed just at the baseline conditions, but were not repeatedly measured during the study period. Thus, studies considering direct and repeated measurements are needed to confirm the longitudinal effects of work schedule pattern and dietary inflammatory potential on the survival of patients. However, the results remained robust after excluding deaths occurring in the first 2 years of follow-up, which can reduce the likelihood of reverse causation.

## Conclusion

Overall, in this prospective cohort study among a nationally representative survey of hypertension patients, we found that shift work increases the risk of all-cause mortality. Moreover, anti-inflammatory dietary pattern can offset the increased mortality risk caused by shift work, but the combination of pro-inflammatory diet and shift work further increases this risk. These findings have implications on further research. The occupational exposure like shift work and daily dietary pattern should be considered jointly in future observational and intervention studies among CVD patients. Moreover, longitudinal studies to assess the dietary inflammatory potential in the survival of hypertensive shift workers or population with similar risk profiles are of urgent need.

## Data Availability

The NHANES data supporting the results of this study are available online through https://wwwn.cdc.gov/nchs/nhanes/Default.aspx.
